# *TBX21* correlates with an immunosuppressive tumor microenvironment and Treg/Th17 imbalance in prostate cancer

**DOI:** 10.3389/fonc.2025.1701148

**Published:** 2026-01-07

**Authors:** Heng Wang, Zhaofei Liu, Xiangjun Xu, Qichao Wang, Pingan Chang, Lei He, Jun Ouyang

**Affiliations:** 1 Department of Urology, The First Affiliated Hospital of Soochow University, Suzhou, China; 2 Department of Urology, Lianyungang Affiliated Hospital of Nanjing University of Chinese Medicine, Lianyungang, Jiangsu, China; 3 Department of Urology, Xuzhou Cancer Hospital, Affiliated Hospital of Jiangsu University, Xuzhou, Jiangsu, China; 4 Department of Urology, Dongtai People Hospital, Yancheng, Jiangsu, China

**Keywords:** prostate cancer, TBX21, Treg, immunosuppression, immune microenvironment

## Abstract

**Background:**

Prostate cancer (PCa) remains a major health burden, and identifying target molecules will provide new research ideas for treating PCa. *TBX21* is a transcriptional factor with critical functions in tumor development, roles of which in PCa were studied herein.

**Methods:**

*TBX21* expression was evaluated by RNA-seq, RT-PCR, Western blot, and immunohistochemistry in PCa tissues and cell lines. Functional assays were performed using LNCaP and 22RV1 cells with *TBX21* knockdown to assess proliferation and apoptosis. Tumor growth and immune alterations were examined in xenograft and humanized immune cell models, while flow cytometry and Western blot were used to characterize immune cell subsets and effector molecules.

**Results:**

*TBX21* expression was significantly elevated in PCa tissues and cell lines. *TBX21* knockdown was associated with reduced proliferation and increased apoptosis *in vitro* and suppressed tumor growth *in vivo*. In humanized xenografts, *TBX21* silencing was accompanied by a decrease in regulatory T cells (Treg) and an increase in Th17 and cytotoxic CD8^+^ T cells, together with enhanced expression of effector molecules (TNF-α, GZMB). Co-culture experiments showed that *TBX21*-deficient tumor cells reduced the induction of CD25^+^Foxp3^+^ Treg cells from activated CD4^+^ T cells.

**Conclusion:**

*TBX21* is associated with tumor progression and an immunosuppressive microenvironment in PCa, underscoring its potential role in modulating the tumor–immune balance.

## Introduction

Cancer represents a significant health concern on a worldwide scale. In accordance with the most recent statistics provided by the American Cancer Society during the year 2022, PCa has become the most prevalent cancer in men with an incidence rate of 27% and the second highest mortality rate at 11% (1). Approximately 81% of new cases diagnosed annually in the United States are clinically localized PCa, while in China, 33% of PCa cases are early-stage patients, with the remainder being late-stage or metastatic patients. The survival rate for late-stage and metastatic patients is extremely low, and the 5-year survival rate is significantly lower than that in developed countries in Europe and America, imposing a heavy burden on public health and the economy (2, 3). Progressive PCa refers to the disease progression in patients with localized PCa after surgical treatment or radiotherapy, or distant metastasis before treatment. The main treatments for PCa include androgen deprivation therapy (ADT), adjuvant chemotherapy, and novel endocrine therapies. Even after radical treatment, 30% to 40% of patients still experience recurrence, and the recurrence rate in high-risk PCa patients exceeds 50% (4). Despite the availability of numerous treatment plans, the progression of the disease ultimately cannot be avoided. Therefore, in-depth research on the genomic changes during the progression of PCa, identification of key target molecules, and elucidation of their mediated molecular mechanisms and biological effects will provide new research ideas for the development of targeted drugs for PCa.

The tumor immune microenvironment (TIME) exerts a vital influence on PCa, where the growth and metastasis of PCa are closely in connection with immune cells, cytokines, growth factors, and extracellular matrix components within the tumor microenvironment (5). In the early stages of PCa, immune cells within the tumor microenvironment, such as T cells, dendritic cells, and natural killer (NK) cells, are capable of recognizing and attacking tumor cells, performing immune surveillance functions. However, as the tumor progresses, malignant cells bypass the immune system’s scrutiny utilizing diverse strategies, such as secreting immunosuppressive factors, inducing apoptosis of immune cells, or altering their phenotype, thereby forming an immunosuppressive microenvironment (6, 7). Additionally, fibroblasts, endothelial cells, and stromal cells within the TIME also participates in the blood vessel formation, penetration, and spreading of neoplasms, which foster the expansion and endurance of malignant cells by releasing cytokines and proteins of the extracellular matrix (8). A deep understanding of the mechanisms by which the tumor immune microenvironment acts in PCa is conducive to the development of new treatment strategies, improving the quality of life and survival rates of patients. Regulatory T cells (Treg), a subset of T lymphocytes, primarily mediate immune suppression within the body by secreting inhibitory cytokines, inducing apoptosis, and directly transmitting inhibitory signals, ultimately achieving the maintenance of homeostasis (9). Treg cells full a crucial function in autoimmune diseases, tumorigenesis, and organ transplantation. In the occurrence and development of tumors, Treg cells aggregate locally in tumors and induce tumor escape by inducing immune suppression (10). Therefore, targeting the clearance or inhibition of Treg cells is a new direction for research to enhance the effect of tumor immunotherapy.

*TBX21*, encodes the transcription factor T-bet, which belongs to the T-box family and contributes significantly to cell differentiation and development, mainly by regulating gene expression to affect the fate of cells (11). *TBX21* is expressed in various cell types, especially in immune cells such as T cells, where it participates in the regulation of immune responses via mediating the expression of specific genes (12). The function of *TBX21* is not limited to immune regulation but also involves the development of tissues such as the heart and skeletal muscle, and its abnormal expression is related to various diseases, such as autoimmune diseases and certain cancers (13). In immune and tumor contexts, *TBX21*/T-bet is a canonical downstream target of interferon-γ–STAT1 signaling and helps imprint Th1-like programs (14–16). In PCa, where androgen-receptor (AR) signaling is a dominant driver, AR has been reported to crosstalk with inflammatory/interferon pathways and to influence tumor–immune interactions (17). Moreover, recent work on Pca lineage plasticity shows that inflammatory and AR-indifferent states can coexist in therapy-resistant disease, which provides a biological rationale to investigate TBX21 in Pca (18, 19). *TBX21* influences the development and function of Treg cells by regulating the expression of specific genes, thus participating in the balance of immune responses, which not only promotes the differentiation and survival of Treg cells but also affects their ability to inhibit other immune cells (20, 21). Peng has reported the role of *TBX21* in PCa (22). However, whether *TBX21* affects the immunosuppressive microenvironment of PCa is not clear. Herein, *TBX21*’s role in PCa immunosuppressive microenvironment was clarified by exploring its impact on the immune repressive property of Treg cells, providing new molecular targets for PCa immunotherapy.

## Materials and methods

### Clinical samples collection, RNA-seq, and survival analysis

Samples were sourced from the Department of urology, Lianyungang Affiliated Hospital of Nanjing University of Chinese Medicine, consisting of PCa tissues and corresponding paracancerous tissues from patients who underwent surgical treatment for PCa and were pathologically diagnosed with PCa. For RNA-seq analysis, three pairs of primary prostate cancer tissues and their matched paracancerous tissues were selected; basic clinicopathological information (age, serum PSA, and Gleason score) was recorded to ensure comparability of the paired samples. Paracancerous tissues (≥5 cm from tumor margin) were collected and histologically confirmed by two independent pathologists to contain no malignant or atypical glands or inflammatory infiltration. Although these tissues may not fully represent normal prostate, they were used as the best available internal control. All specimens applied here were achieved with the patients’ informed consent and were approved by the Ethics Committee of Lianyungang Affiliated Hospital of Nanjing University of Chinese Medicine (No: 2023-KY-105).

PCa and paracancerous tissues were sent to BioMarker (China) for RNA-sequence to determine the differentially expressed genes. Total RNA was extracted with TRIzol (Invitrogen) and RNA integrity was assessed by Agilent 2100 Bioanalyzer (RNA integrity number ≥ 7.0). Poly(A)-selected libraries were prepared following the manufacturer’s protocol (Illumina) and sequenced on an Illumina NovaSeq 6000 platform to generate 150-bp paired-end reads. Raw reads were quality-filtered to remove adapters and low-quality bases, and clean reads were aligned to the human reference genome (GRCh38) using STAR. Gene-level counts were obtained with featureCounts and normalized using DESeq2. Batch effects across samples were evaluated and, when necessary, adjusted using the sva package. Differentially expressed genes were defined as those with |log2(fold change)| ≥ 1 and Benjamini–Hochberg adjusted P (FDR) < 0.05. A comparative analysis was executed using Venn diagrams, leading to the discovery of a set of 3806 genes correlated with the prognosis of PCa. The RNA-seq dataset has been deposited in the GEO repository so that raw and processed data will be publicly available (Accession number.GSE310339).

### Immunohistochemical assay

Tumor or normal tissues were fixed in 10% neutral formalin for 24 hours, then subjected to a series of alcohol solutions of varying concentrations to gradually replace the water content within the tissues. Subsequently, a clarification process was carried out, utilizing solvents such as xylene to remove lipids from the tissues. Following this, an infiltration with paraffin was performed, immersing tissues in paraffin. The hardened tissues were then sectioned into slices with a thickness of 3–5 micrometers. Paraffin sections were routinely deparaffinized to water and cultured with 3% hydrogen peroxide in deionized water for 10 to 30 minutes to inactivate endogenous peroxidase activity. After being blocked with goat serum for 15 to 30 minutes, sections were incubated with the T-bet or Ki67 primary antibody (1:400, CST, United States) at 37 °C for 2 to 3 hours. The following day, the sections were treated with a secondary antibody (1:400, CST, United States) conjugated to horseradish peroxidase (HRP) and incubated for 30 minutes. The reaction was visualized using a chromogen (DAB), and the duration of the reaction was controlled under a microscope. Sections were then counterstained with hematoxylin, dehydrated in a graded series, cleared, and mounted with neutral balsam for staining. Levels of T-bet were observed under a microscope (Zeiss, Germany).

### RT-PCR assay

RNAs were isolated from PCa samples and healthy samples utilizing the Trizol reagent (Thermo, United States). The density and integrity of the nucleic acid were ascertained with an ultra-sensitive nucleic acid and protein quantification device. Subsequently, the nucleic acid was converted into cDNA through a reverse transcription kit (Thermo, United States). Subsequent to this, qPCR with real-time fluorescence detection was executed using the SYBR Premix Ex Taq II kit (Takara, Japan) on a PCR machine (BD, United States). Levels of relevant genes were determined utilizing the 2^-△△Ct^ relative quantification method.

### Western blot analysis

Total protein contents from cellular or oncological samples were obtained using a protein extraction kit (ZIKER, China). The measurement of the harvested overall protein density was accomplished utilizing a BCA quantification set (Solarbio, China). Protein samples were subjected to SDS-PAGE gel electrophoresis. After the electrophoresis was completed, proteins were conveyed to a polyvinylidene fluoride film. The film was then occluded with a 5% defatted milk solution for the duration of 2 hours in the dark. Primary antibody dilutions (T-bet, STAT1, TNF-α, GZMB, Perforin, Bim, BID, BAD at a ratio of 1:1000; GAPDH at a ratio of 1:2000; CST, United States) were appended and allowed to interact with the sample throughout the night at a temperature of 4°C. Following this, solutions of secondary immunoglobulins (1:2000, CST, United States) were applied were added and incubated for 1 hour. Protein levels were analyzed using a protein gel imaging system and Image J software.

### Cells and treatments

RWPE-1 cells and PCa cell lines (LNCaP, 22RV1, PC3, DU145) were purchased from ATCC (United States). RWPE-1 cells were cultured in Eagle’s Minimum Essential Medium, while PCa cells were cultured in Ham’s F12K medium. Culturing mediums were supplemented with 10% FBS and the culture conditions were 5% CO2 and 37°C. To silence *TBX21* in PCa cells, cells were transfected with the lentivirus supplemented with two independent shRNA targeting *TBX21* (sh-*TBX21*), with sh-NC as a negative control. After 48 h transfection, the silence efficacy was verified by Western blot, and the shRNA with stronger knockdown efficiency was used for subsequent experiments.

### CCK-8 assay

The cell suspension digested with trypsin was added to a 96-well plate and then transferred to a cell culture incubator for cultivation. Following treatments and transfections, cells were stimulated with 10 μL of CCK-8 solution and then placed back into the cell culture incubator for an additional 3 hours. The viability of the cells was determined by measuring the absorbance (450nm) using a microplate reader (MD, United States).

### EdU assay

Cells were inoculated into a 24-well culture dish, and post 24 hours, a 50 μmol/L 5-ethynyl-2’-deoxyuridine (EdU) reagent was incorporated to sustain the cultivation. Following a further 2 hours, the cellular entities underwent rinsing, became rigidified, and subsequently were scrutinized and visually documented via a luminescence optical instrument (Zeiss, Germany). The EdU incorporation rate was evaluated employing Image J software, determined as the proportion of EdU-positive cellular entities to the overall cellular count and then multiplied by 100% to express the result as a percentage.

### Apoptosis analysis

Following treatments, cells were digested and neutralized with complete medium, and the cell pellet was collected by centrifugation at 1000 rpm. The cell pellet was resuspended in 500 μL of binding buffer, followed by introducing 50 μL Annexin V-FITC and 10 μL PI. After incubation in the dark for 15 minutes, the number of PI-positive cells was detected using a FACS Canto II flow cytometer (BD, United States).

### Xenograft model in nude mice

1×10^7^ 22RV1 cells were collected and inoculated subcutaneously into nude mice. When the volume of tumor reached 800 mm^3^, 2 groups were divided: Sh-NC and sh-*TBX21*. In the sh-NC and sh-*TBX21* groups, tumor-bearing mice were injected with lentivirus supplemented with sh-NC and sh-*TBX21* via the tail vein, respectively, followed by recording the growth of tumor every day. On the last day, tumor tissues were collected for subsequent assays. This study was approved by the Animal Ethics Committee of Bestcell Model Biological Center (approval number: BSMS 2024-07-23B).

### HE staining

Tumor tissues were excised for the processes of cleansing, desiccation, encasement in paraffin, and slicing. The paraffin slices were subjected to a heating procedure, followed by the removal of paraffin and a rehydration sequence. The slices to which distilled water had been applied were then immersed in a hematoxylin staining solution for a duration of 3 minutes, subjected to a hydrochloric acid-ethanol solution for differentiation for a period of 15 seconds, gently rinsed with water, briefly returned to a bluing agent for 15 seconds, washed with flowing water, imbued with eosin for 3 minutes, cleansed with running water, underwent a series of desiccation stages, were rendered transparent, and were finally mounted and subjected to microscopic examination using an optical microscope (Zeiss, Germany).

### TUNEL staining assay

Tumor tissue samples were fixed in 4% paraformaldehyde for at least 4 hours, then permeated overnight at 4 °C with 30% sucrose solution, and the permeated blocks were frozen in OCT compound and cut into 5-micron thick serial sections, attached to precooled microscope slides. Sections were subsequently treated with 0.1% proteinase K for 10 minutes at 37 °C to promote DNA digestion and incubated with TUNEL reaction mixture in a wet box for 1 hour to label the broken ends of apoptotic cell DNA. Sections were then incubated with fluorescently labeled anti-fluorescein antibody for 30 minutes to amplify TUNEL signals, and finally the number of TUNEL-positive cells was observed and analyzed using confocal microscopy (Zeiss, Germany) or fluorescence microscopy to quantitatively assess the level of apoptosis in tumor tissues of each group.

### The establishment of xenograft model in nude mice with human immune system

Peripheral blood of a single healthy human donor (age 29 years) with no history of autoimmune, inflammatory, or malignant disease. PBMCs were isolated utilizing the Ficoll-Paque density gradient sedimentation technique. Approximately 1×10^7^ PBMCs were injected into the tail vein of male athymic nude mice (6–8 weeks old, 18–22 g) to establish a human immune system. Mice were maintained for 2–3 weeks post-engraftment, a time window reported to achieve stable human T-cell reconstitution and minimal GVHD development. Subsequently, 1×10^7^ 22RV1 cells were collected and inoculated subcutaneously into nude mice with human immune system. When the volume of tumor reached 800 mm^3^, mice were randomly assigned to receive tail-vein injection of lentivirus carrying sh-NC or sh-*TBX21*. Tumor growth was monitored and recorded daily by investigators blinded to treatment allocation.

### Immunofluorescence staining

Tumor sections (4 µm) from humanized 22RV1 xenografts were deparaffinized, subjected to antigen retrieval, and blocked with 5% BSA. Sections were incubated overnight at 4 °C with antibodies against T-bet (CST #13232), EpCAM (Abcam ab71916), or CD3 (Abcam ab16669), followed by Alexa Fluor-conjugated secondary antibodies (Invitrogen). Nuclei were counterstained with DAPI, and images were acquired on a fluorescence microscope. T-bet fluorescence intensities in EpCAM^+^ and CD3^+^ regions were quantified using ImageJ.

### The identification of CD4^+^ T and CD8^+^ T cell ratio in tumor tissues

The tumor tissue was pulverized and strained, ensuing that the liquid residue was spun at a velocity of 1,500 rpm for the duration of 5 minutes. A two-milliliter volume of erythrocyte dissolution solution was dispensed and the blend was processed for half a decennium, complemented by an infusion of a PBS quantity threefold the size of the initial for neutralization purposes. Post a subsequent 5-minute spin at 1,500 rpm, the sediment was re-liquefied and a quantity of 1×10^6^ cells per specimen was selected for exterior complexion decoration. A microliter measure of anti-CD16/CD32 immunoglobulin (CST, United States) was appended to each container to impede the Fc receptors situated on the cellular exterior, with the blend being set aside for a decadal interval at a temperature of 4 °C. Following this, immunoglobulins specific for CD3 conjugated with FITC (CST, United States) were integrated into the darkness at 4 °C for the span of 30 minutes, trailed by the incorporation of immunoglobulins specific for CD4 conjugated with PECy7 and CD8 conjugated with PE (CST, United States) for a 30-minute period of incubation. After a 5-minute spin at 1,500 rpm, the cellular entities were re-suspended in a 200-microliter PBS solution and proceeded to cytometric scrutiny using a flow cytometer (BD, United States). The cytometric numerical values were charted and interpreted for the relative frequencies of CD4^+^ T lymphocytes and CD8^+^ T lymphocytes employing FlowJo analytical software.

### The identification of Treg/Th17 ratio in tumor tissues

To determine the ratio of Treg and Th17 cells in tumor tissues, CD4^+^ T cells were isolated using the sorting flow cytometry (BD, United States). Subsequently, IL17A-FITC and Foxp3-PE antibodies (CST, United States) were loaded for 30 min incubation, followed by resuspension and loaded onto the flow cytometry (BD, United States) to determine the ratio of Treg (Foxp3^+^CD4^+^ T) and Th17 (IL17A^+^CD4^+^ T) cells using FlowJo software.

### The identification of cytotoxic CD8^+^ T cell ratio in tumor tissues

To determine the cytotoxic function of CD8^+^ T cells in tumor tissues, CD8^+^ T cells were isolated using the sorting flow cytometry (BD, United States). Subsequently, TNF-α-PECy7 and GZMB-PE antibodies (CST, United States) were added for 30 min incubation, followed by resuspension and loaded onto the flow cytometry (BD, United States) to determine the ratio of TNF-α^+^CD8^+^ T and GZMB^+^CD8^+^ T cells using FlowJo software.

### The identification of immunosuppressive CD4^+^ T and CD8^+^ T cell ratio in tumor tissues

CD4^+^ T cells and CD8^+^ T cells were isolated using the sorting flow cytometry (BD, United States). PD-1-FITC, Tim-3-PE, and Lag-3-PECy7 antibodies (CST, United States) were loaded into CD4^+^ T cells and CD8^+^ T cells, respectively. The mixture was incubated in the dark at 4 °C for 30 minutes. After centrifugation at 1,500 rpm for 5 minutes, cells were resuspended in 200 μl of PBS and analyzed on a flow cytometer (BD, United States). The flow cytometry data were plotted and analyzed for the proportions of PD-1^+^CD4^+^ T cells, Tim-3^+^CD4^+^ T cells, Lag-3^+^CD4^+^ T cells, PD-1^+^CD8^+^ T cells, Tim-3^+^CD4^+^ T cells, and Lag-3^+^CD8^+^ T cells using FlowJo software.

### The identification of Treg cell ratio in the co-culture system

Naive CD4^+^T cells were isolated from human PBMC using the sorting flow cytometry (BD, United States), followed by stimulated by CD3/CD28 functional antibody (CST, United States) for activation. Activated CD4^+^ T cells were co-cultured with sh-NC or sh-*TBX21* transfected LNCaP and 22RV1 cells in a Transwell system (0.4-μm pore size; Corning, United States). After 24 h of co-culture, CD4^+^ T cells in the upper chamber were collected, stained with CD25-FITC and Foxp3-PE antibodies (CST, United States) for 30 min in the dark, followed by loading to the flow cytometry (BD, United States) to determine the ratio of Treg (CD25^+^Foxp3^+^) cells.

### Conditioned medium assay

Supernatants from sh-NC or sh-*TBX21* LNCaP and 22RV1 cells cultured for 48 h were collected, centrifuged, and filtered through 0.22-μm membranes. Activated CD4^+^ T cells were cultured in 50% conditioned medium for 48 h with or without neutralizing antibodies against TGF-β (10 μg/mL). Treg (CD25^+^Foxp3^+^) proportions were then determined by flow cytometry.

### Statistical analysis

The empirical outcomes were processed utilizing the SPSS 25.0 computational suite. Data are presented as the mean ± standard deviation (x ± s). In instances where the numerical sets exhibited a regular distribution and uniform variability, the Student’s t-test was employed for pairwise assessments, while differences among multiple groups were evaluated using a one-way analysis of variance (ANOVA). Tumor growth curves were analyzed by two-way repeated-measures ANOVA with Greenhouse–Geisser correction. Flow cytometry results, expressed as proportions, were arcsine-square-root transformed prior to analysis, and false discovery rate (FDR) correction was applied using the Benjamini–Hochberg method for multiple-marker comparisons. A P-value < 0.05 was considered statistically significant. All visual representations were rendered using GraphPad Prism 9.4.1, with individual data points overlaid on mean ± SD bars.

## Results

### *TBX21* was upregulated in PCa tissues and cell lines

**Figure 1:**
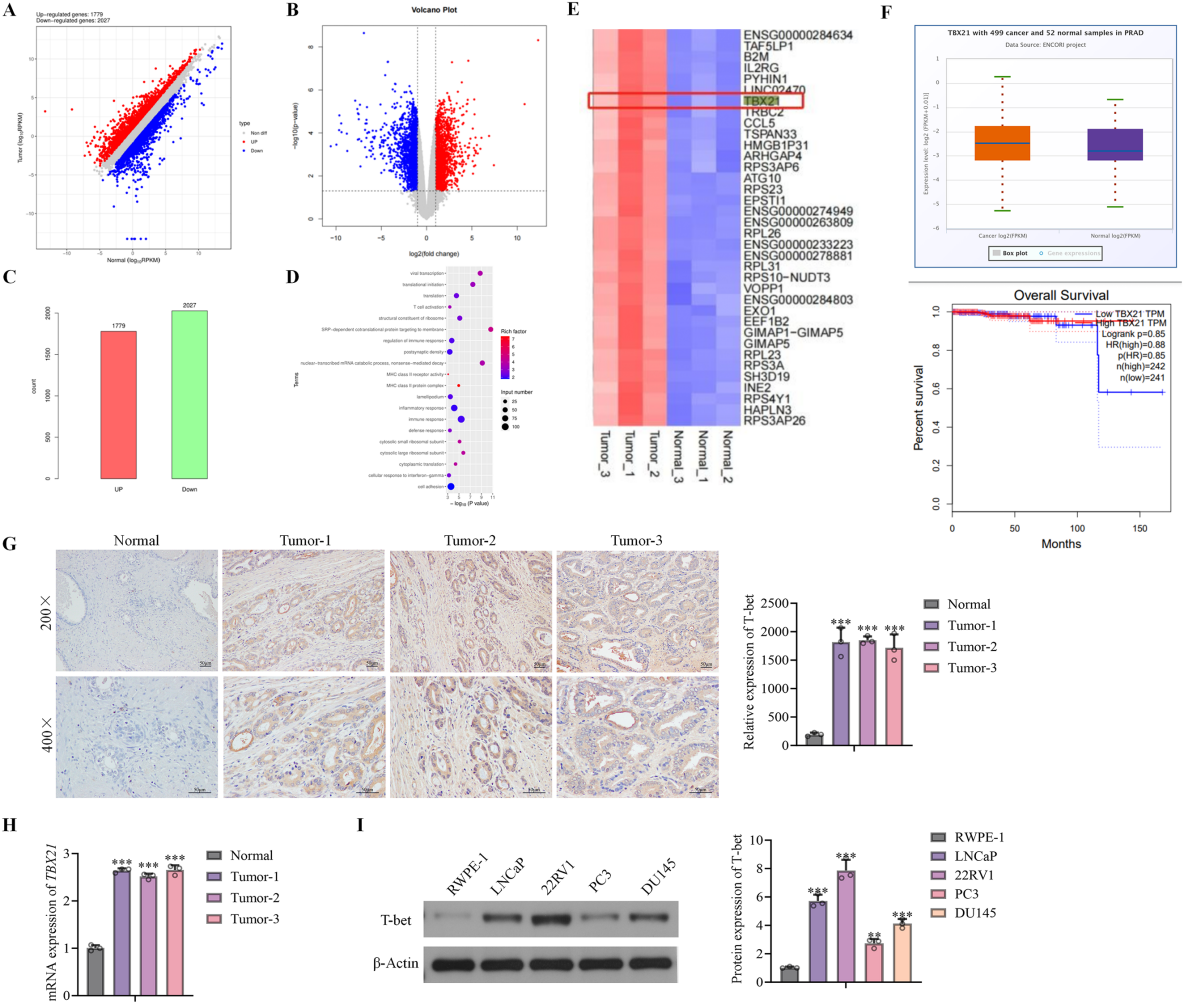
*TBX21* is upregulated in PCa. **(A)** Differentially expressed genes (DEGs) between PCa and paracancerous tissues. Red spots represented upregulated genes, and blue spots represented downregulated genes. Gray spots indicated no significant difference. **(B)** Volcano plot of DEGs between PCa and paracancerous tissues (fold change > 2, p < 0.05). **(C)** Summary of 1,779 upregulated and 2,027 downregulated genes in PCa tissues compared with paracancerous tissues. **(D)** Pathway enrichment analysis of DEGs. **(E)** Heatmap of representative DEGs. **(F)** Independent validation in TCGA-PRAD: (top) TBX21 transcript levels in tumors vs normal; (bottom) overall-survival Kaplan–Meier by TBX21 high vs low. G.Immunohistochemical detection of T-bet in normal and tumor tissues. **(H)** RT–PCR analysis of *TBX21* mRNA expression in normal and tumor tissues. **(I)** Western blot analysis of T-bet levels in RWPE-1, LNCaP, 22RV1, PC3, and DU145 cells. Data are presented as mean ± SD, with individual data points overlaid. Statistical significance was determined by one-way ANOVA; n = 3 independent experiments. **p < 0.01, ***p < 0.001 *vs.* normal tissue or RWPE-1 cells.

To explore the function of *TBX21* in PCa, RNA-seq was performed on PCa tissues and normal tissues, in which 1779 genes found upregulated and 2027 genes found downregulated. Comparing to normal tissues, *TBX21* was markedly upregulated in PCa tissues (**Figures 1A-E**). Subsequently, IHC staining and RT-PCR of *TBX21* in normal and tumor tissues was conducted for validation. We found that Microarray data *TBX21* levels were sharply boosted in PCa tumor tissues (**Figure 1E**). Analysis of the TCGA-PRAD cohort further confirmed higher TBX21 transcript levels in tumors versus normal prostate; the corresponding overall-survival curve is shown in **Figure 1F**. In our cohort, T-bet protein was markedly increased in tumor tissues by IHC with quantitative scoring (**Figure 1G**), and TBX21 mRNA was higher in tumors by qRT-PCR (**Figure 1H**). Furthermore, *TBX21* protein levels were evaluated in a panel of prostate cell lines, including normal prostate epithelial RWPE-1, AR-positive (LNCaP, 22RV1), and AR-negative (PC3, DU145) cancer cells. As shown in **Figure 1H**, *TBX21* expression was markedly elevated in prostate cancer cell lines compared with RWPE-1, with the highest levels observed in AR-positive cells. Thus, LNCaP and 22RV1 cells were applied in subsequent assays.

### *TBX21* facilitated proliferation and repressed apoptosis in PCa cells

**Figure 2:**
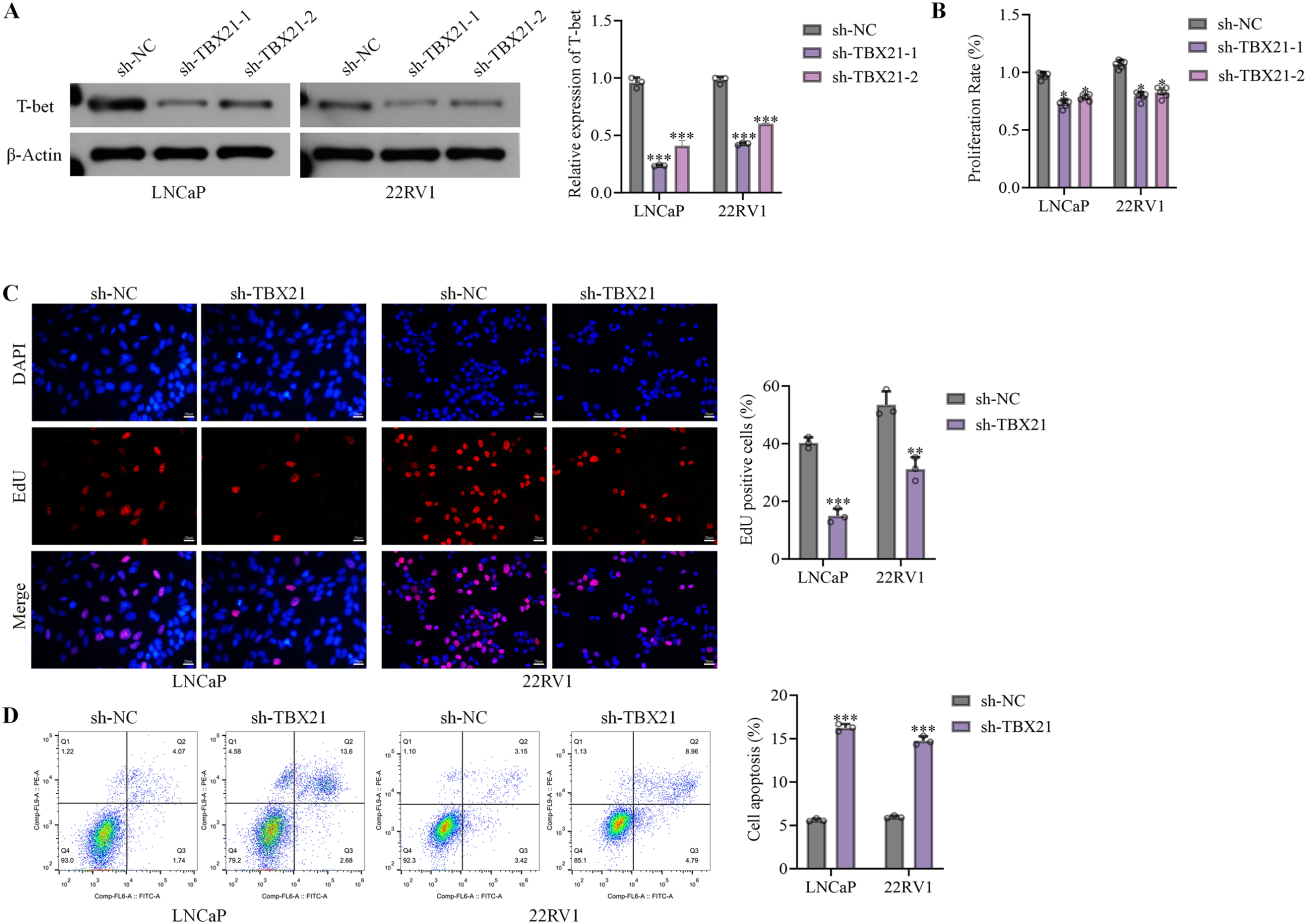
Effects of *TBX21* silencing on proliferation and apoptosis of PCa cells. LNCaP and 22RV1 cells were transfected with lentiviruses expressing two independent shRNAs targeting *TBX21* (sh-*TBX21–*1 and sh-*TBX21*-2) or a negative control (sh-NC). **(A)** The silence efficacy of *TBX21* was validated by Western blot. **(B)** Cell viability was assessed by CCK-8 assay using both shRNA constructs. sh-*TBX21–*1 was selected for subsequent assays. The proliferation was determined by EdU staining assay **(C)** and the apoptosis was analyzed by flow cytometry **(D)**. Data are presented as mean ± SD, with individual data points overlaid. Statistical significance was determined by one-way ANOVA; n = 3 independent experiments. ^*^p<0.05, ^**^p<0.01, ^***^p<0.001 *vs*. sh-NC.

To silence *TBX21* in LNCaP and 22RV1 cells, two independent shRNA constructs targeting non-overlapping regions of *TBX21* (sh-*TBX21*#1 and sh-*TBX21*#2) and a negative control (sh-NC) were transfected into LNCaP and 22RV1 cells using lentivirus. Both shRNAs markedly reduced T-bet level as confirmed by Western blot (**Figure 2A**). Consistent with each other, sh-*TBX21*#1 and sh-*TBX21*#2 both significantly suppressed cell viability as determined by CCK-8 assay (**Figure 2B**). Based on comparable knockdown efficiency and phenotypic trends, sh-*TBX21*#1 was selected for subsequent assays, including EdU staining and Annexin V analysis, which further demonstrated reduced proliferation and increased apoptosis in *TBX21*-silenced cells (**Figures 2C, D**). These findings indicate that *TBX21* promotes the proliferative and anti-apoptotic capacities of PCa cells *in vitro*.

### *TBX21* enhanced *in vivo* growth of 22RV1 xenograft model

**Figure 3:**
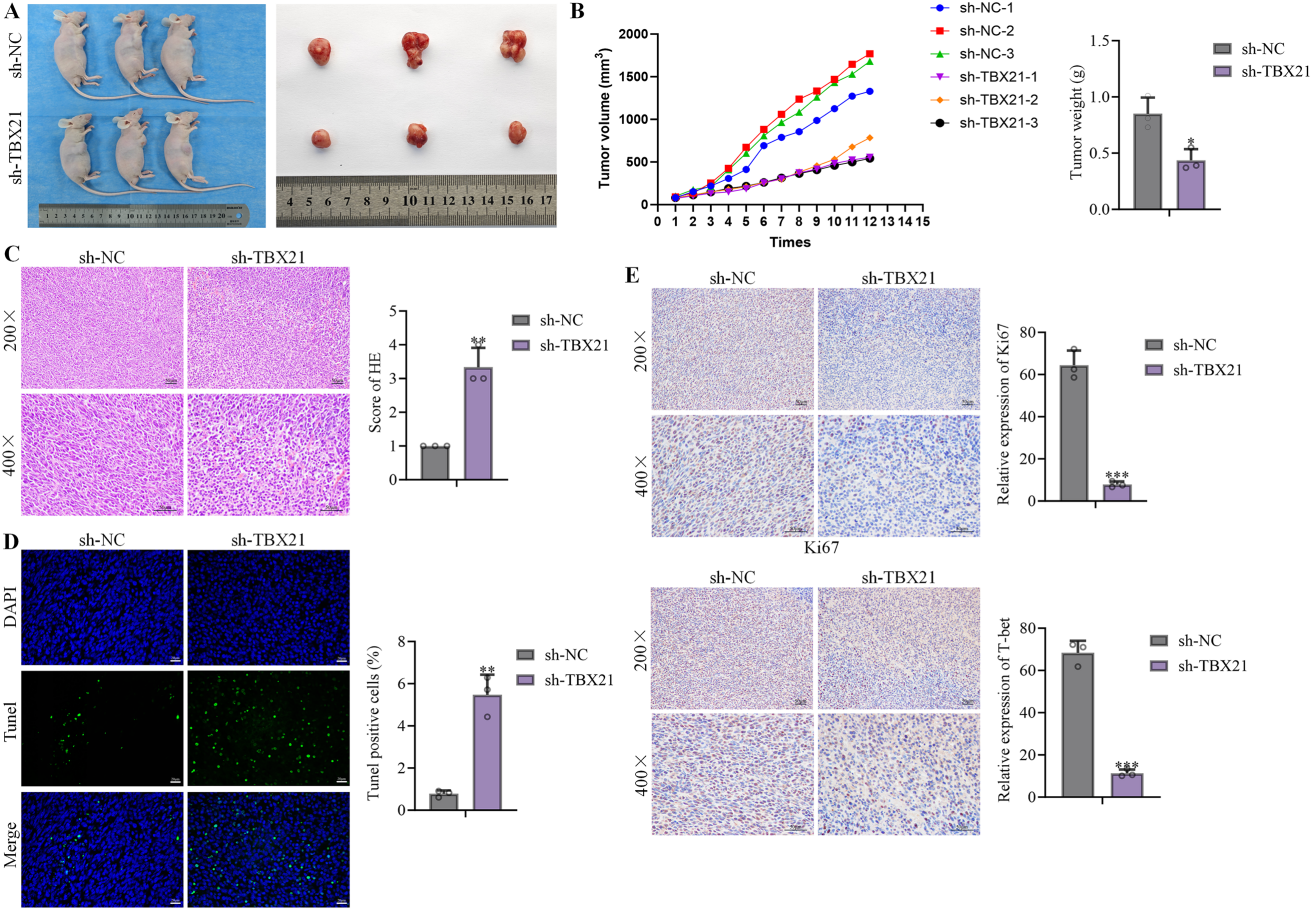
*TBX21* enhanced *in vivo* growth of 22RV1 xenograft model. A xenograft model of 22RV1 cells was established, followed by injection of sh-NC and sh-*TBX21*, respectively. **(A)** Images of tumors were presented. **(B)** Tumor volumes were recorded to draw the tumor growth curve and tumor weights were weighed. **(C)** HE staining was utilized to determine the pathological changes in tumor tissues. **(D)** TUNEL staining was utilized to check the apoptotic status in tumor tissues. **(E)** Ki67 and T-bet expressions in tumor tissues were detected by immunohistochemical assay. Data are presented as mean ± SD, with individual data points overlaid. Tumor growth curves were analyzed by two-way repeated-measures ANOVA with Greenhouse–Geisser correction; other comparisons were evaluated by one-way ANOVA. n = 6 mice per group. ^**^p<0.01, ^***^p<0.01 *vs*. sh-NC.

A xenograft model of 22RV1 cells was established, followed by injection of sh-NC and sh-*TBX21*, respectively. The tumor volume and tumor weight were sharply repressed by sh-*TBX21* (**Figures 3A, B**). Moreover, no significant pathological changes were observed in tumor tissues of sh-NC injected mice. However, loose tissues and infiltrated inflammatory cells were observed in tumor tissues of sh-*TBX21* injected mice (**Figure 3C**). Moreover, TUNEL-stained tumor cells were sharply increased in tumor tissues by the injection of sh-*TBX21*, accompanied by downregulated Ki67 and *TBX21* (**Figures 3D, E**).

### *TBX21* regulated the Th17/Treg balance and cytotoxic function of CD8^+^ T cells in 22RV1 xenograft nude mice model with human immune system

**Figure 4:**
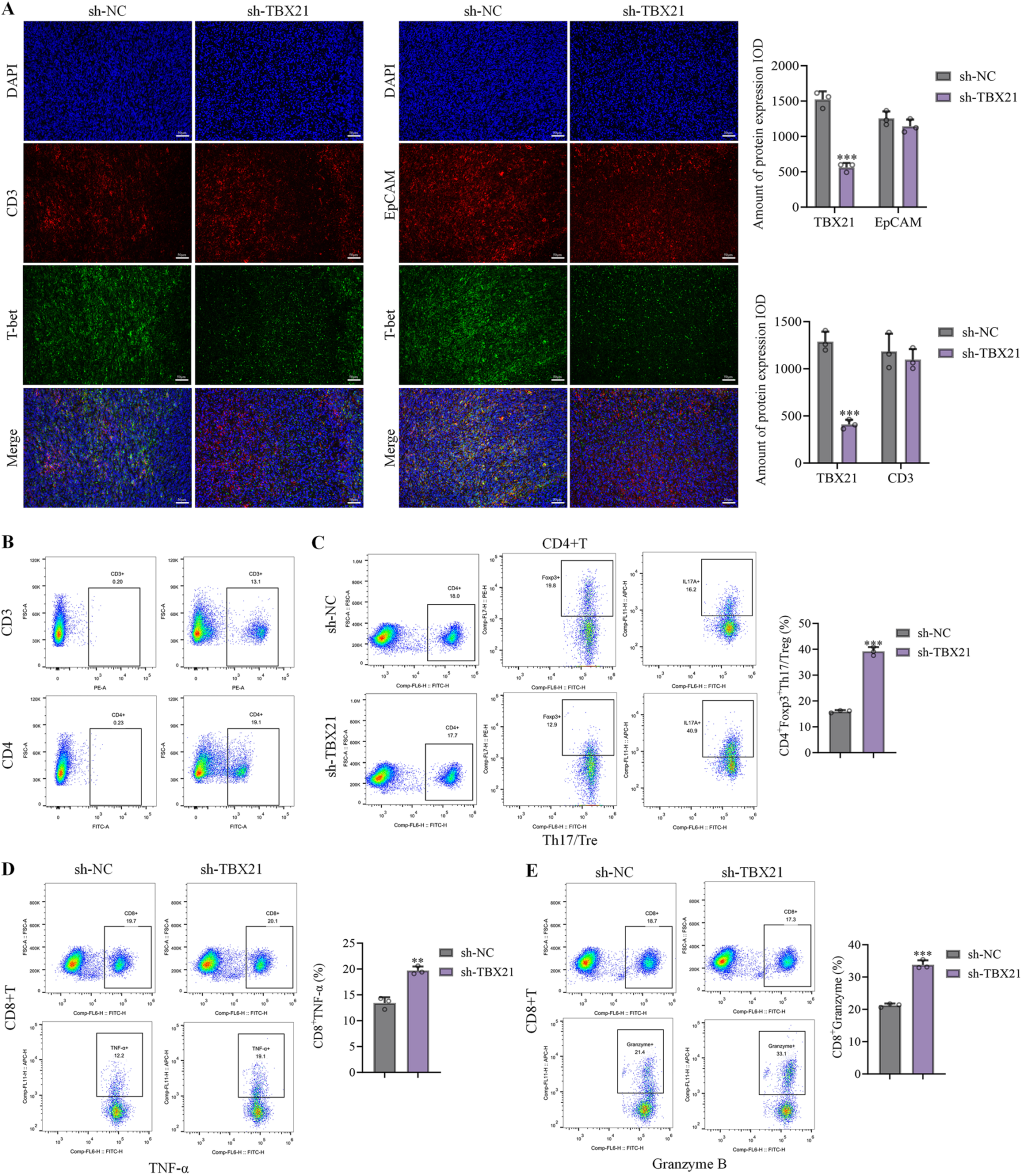
*TBX21* regulated the Th17/Treg balance and cytotoxic function of CD8^+^ T cells in 22RV1 xenograft nude mice model with human immune system. Human PBMC and 22RV1 cells were injected into nude mice to construct the 22RV1 xenograft nude mice model with human immune system, followed by the injections of sh-NC or sh-*TBX21*. **(A)** Double immunofluorescence staining of tumor sections for T-bet together with EpCAM or CD3 to assess cellular localization of T-bet expression. Scale bar = 50 μm. **(B)** The proportion of CD4^+^ T cells in tumor tissues was determined by flow cytometry. **(C)** The proportion of Th17/Treg in tumor tissues was determined by flow cytometry. D-E. The proportion of TNF-α^+^CD8^+^ T and GZMB^+^CD8^+^ T cells in tumor tissues was determined by flow cytometry. Flow cytometry data are shown as representative gating plots together with scatter plots summarizing individual mice, with horizontal bars indicating mean ± SD. Statistical comparisons were performed using one-way ANOVA after arcsine transformation of proportional data; n = 6 mice per group. ^*^p<0.05, ^**^p<0.01, ^***^p<0.001 *vs*. sh-NC.

Human PBMC and 22RV1 cells were injected into nude mice to construct the 22RV1 xenograft nude mice model with human immune system, followed by the injections of sh-NC or sh-*TBX21*. To verify that *TBX21* knockdown occurred specifically within tumor epithelial cells, we performed double immunofluorescence staining for *TBX21* with EpCAM (tumor epithelial marker) and CD3 (T-cell marker). *TBX21* expression was markedly reduced in EpCAM^+^ regions of sh-*TBX21* tumors, whereas no significant difference was observed in CD3^+^ immune cell regions, indicating tumor cell–specific knockdown efficiency (**Figure 4A**). Functionally, the ratio of CD4^+^ T cells was notably increased by following sh-*TBX21* injection (**Figure 4B**). Furthermore, Th17/Treg ratio was sharply enhanced in tumor tissues following sh-*TBX21* injection (**Figure 4C**). Furthermore, percentages of TNF-α^+^CD8^+^ Tand GZMB^+^CD8^+^ T cells in tumor tissues were notably increased by sh-*TBX21* (**Figures 4D, E**), indicating a shift toward a more active antitumor immune microenvironment. Complete gating strategies and representative flow plots for Treg and Th17 analyses are provided in **Supplementary Figure S1**.

### *TBX21* showed no influence on the immune checkpoint in CD4^+^ T and CD8^+^ T cells in 22RV1 xenograft nude mice model with human immune system

**Figure 5:**
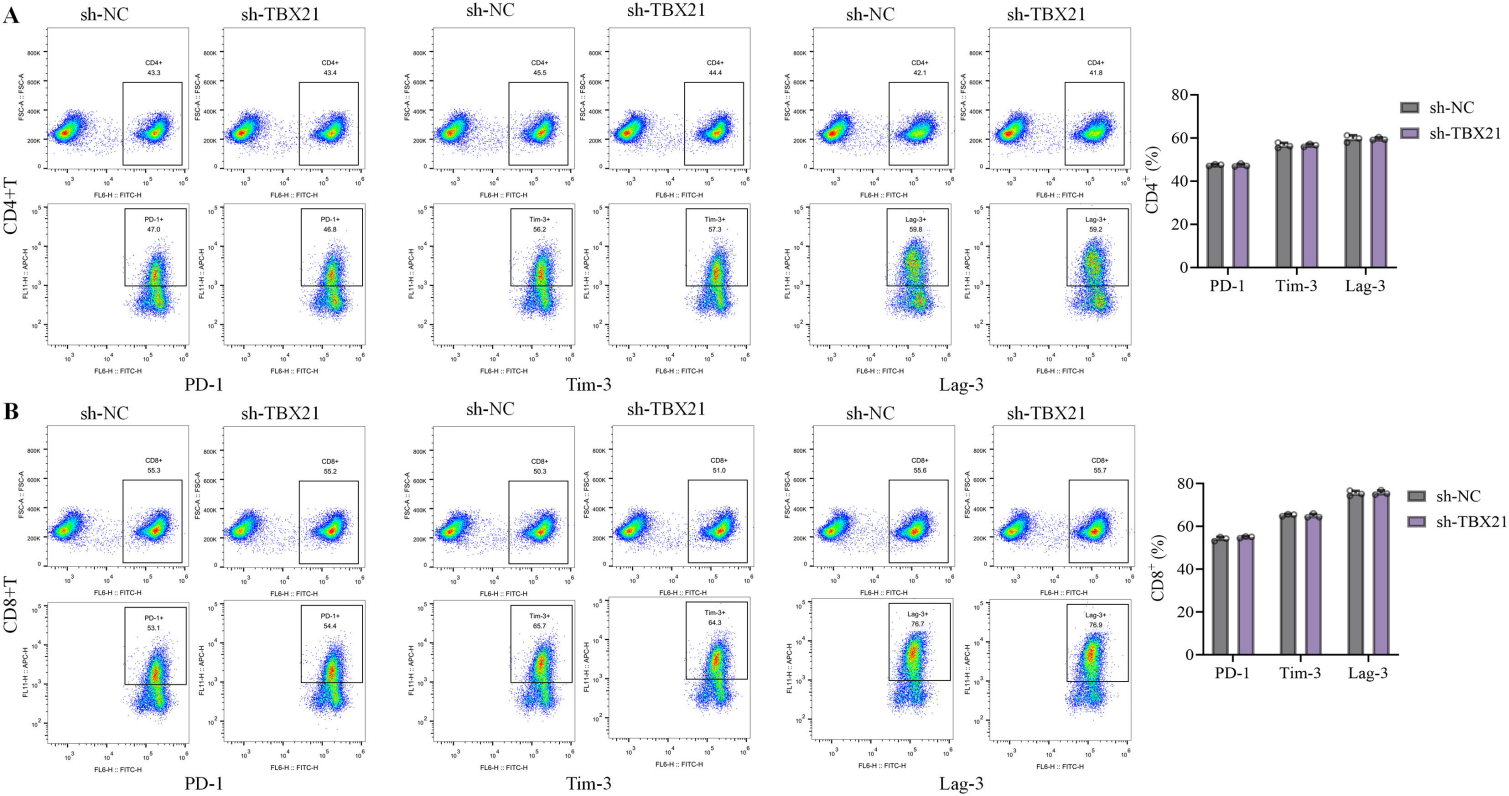
*TBX21* showed no influence on the immune checkpoint in CD4^+^ T and CD8^+^ T cells in 22RV1 xenograft nude mice model with human immune system. **(A)** Percentages of PD-1^+^CD4^+^ T cells, Tim-3^+^CD4^+^ T cells, and Lag-3^+^CD4^+^ T cells were determined by flow cytometry. **(B)** Percentages of PD-1^+^CD8^+^ T cells, Tim-3^+^CD8^+^ T cells, and Lag-3^+^CD8^+^ T cells were determined by flow cytometry. Flow-cytometry results are shown as representative gating plots together with scatter plots summarizing individual mice, with horizontal bars indicating mean ± SD. Statistical comparisons were performed using one-way ANOVA after arcsine transformation of proportional data; n = 6 mice per group.

Subsequently, the influence of sh-*TBX21* on the immune checkpoint in CD4^+^ T and CD8^+^ T cells was evaluated. Following the injection of sh-*TBX21*, no significant changes were observed on the clusters of PD-1^+^CD4^+^ T cells, Tim-3^+^CD4^+^ T cells, Lag-3^+^CD4^+^ T cells, PD-1^+^CD8^+^ T cells, Tim-3^+^CD8^+^ T cells, and Lag-3^+^CD8^+^ T cells in tumor tissues (**Figures 5A, B**).

### *TBX21* was associated with Treg differentiation from activated CD4^+^T cells

**Figure 6:**
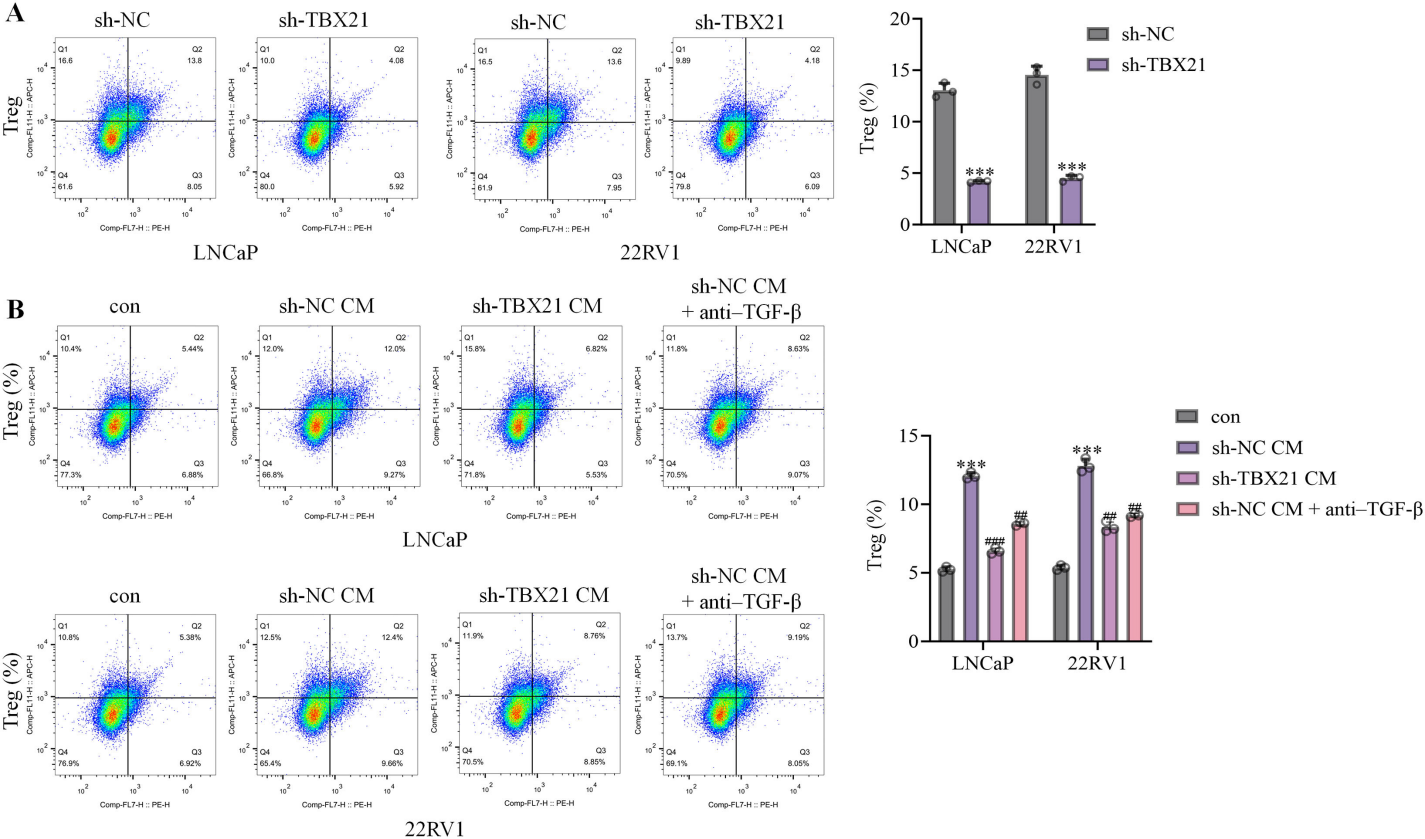
*TBX21* facilitated Treg differentiation in activated CD4^+^ T cells. **(A)** Naive CD4^+^T cells were activated by CD3/CD28 functional antibody, followed by co-cultured in a Transwell system with sh-NC or sh-*TBX21* transfected LNCaP cells or 22RV1 cells. Percentages of CD25^+^Foxp3^+^ Treg cells in activated CD4^+^T cells were determined by flow cytometry. ^***^p<0.001. **(B)** To evaluate soluble mechanisms, conditioned medium (CM) from sh-NC or sh-*TBX21* 22RV1 cells was applied to activated CD4^+^ T cells, with or without TGF-β neutralizing antibody. Treg differentiation was analyzed by flow cytometry. Statistical comparisons were performed using one-way ANOVA after arcsine transformation; n = 3 independent experiments. ^***^p<0.001 *vs.* con, ^###^p<0.001, ^##^p<0.01 *vs.* sh-NC CM.

To explore the potential influence of *TBX21* on Treg generation, naive CD4^+^T cells were activated by CD3/CD28 functional antibody, followed by co-cultured in a Transwell system with sh-NC or sh-*TBX21* transfected LNCaP cells or 22RV1 cells. The proportion of CD25^+^Foxp3^+^ Treg cells among activated CD4^+^ T cells was markedly reduced when co-cultured with *TBX21*-silenced tumor cells compared with sh-NC controls (**Figure 6A**). To examine whether soluble factors contributed to this effect, we performed CM assays. Supernatants from sh-NC or sh-*TBX21* 22RV1 cells were applied to activated CD4^+^ T cells. CM from sh-NC cells promoted Treg differentiation, whereas CM from *TBX21*-silenced cells exhibited a markedly reduced Treg-inducing effect; importantly, neutralization of TGF-β partially reversed the Treg induction by control CM (**Figure 6B**). These findings support a soluble-factor–dependent mechanism, with TGF-β contributing to *TBX21*-linked modulation of Treg differentiation.

### *TBX21* influenced levels of anti-tumor factors in 22RV1 xenograft nude mice model with human immune system

**Figure 7:**
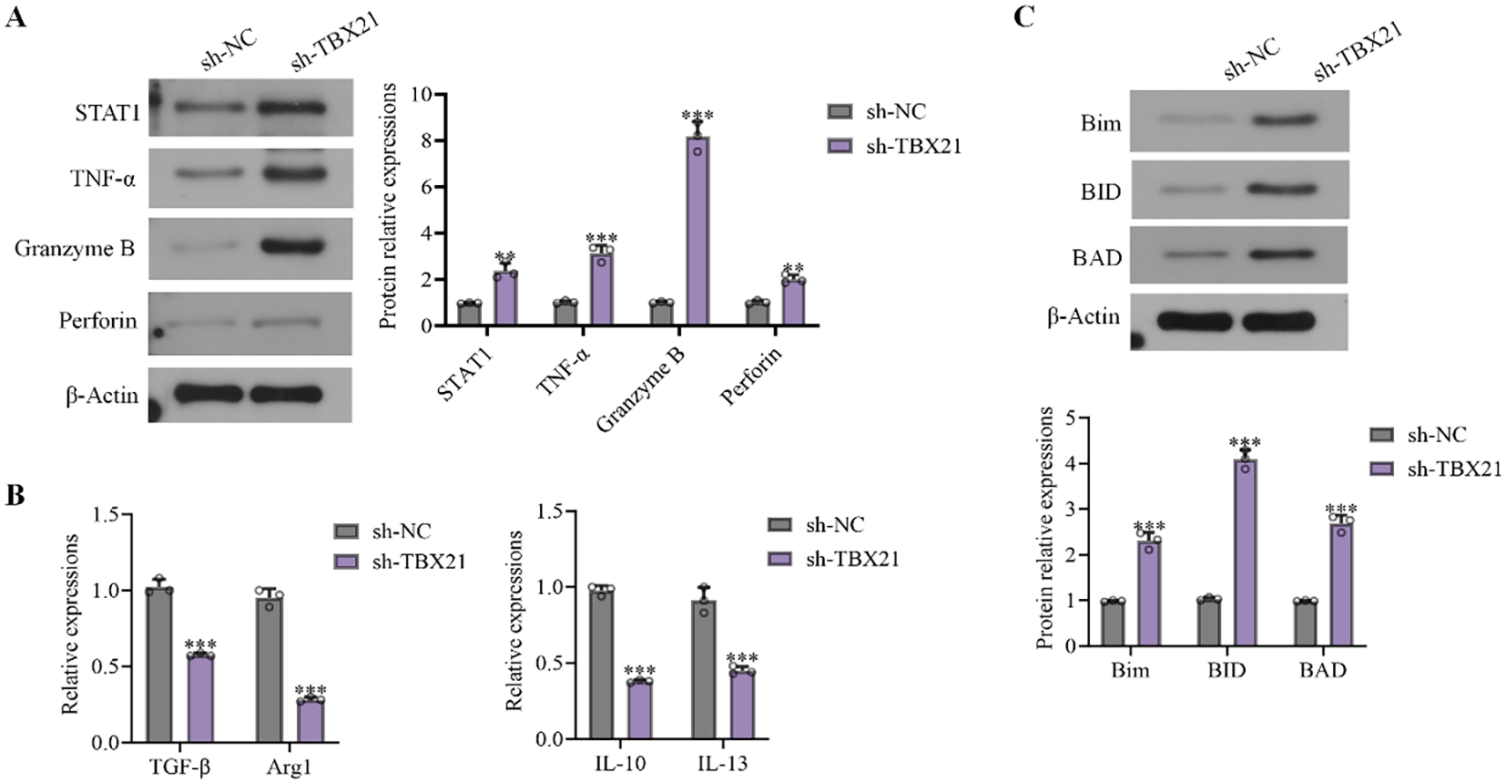
*TBX21* influenced levels of anti-tumor factors in 22RV1 xenograft nude mice model with human immune system. Human PBMC and 22RV1 cells were injected into nude mice to construct the 22RV1 xenograft nude mice model with human immune system, followed by the injections of sh-NC or sh-*TBX21*. **(A)** Expressions of STAT1, TNF-α, GZMB, and Perforin in tumor tissues were detected by Western blot. **(B)** TGF-β, IL-10, IL-13, and Arg1 levels in tumor tissues were determined by Western blot. **(C)** Bim, BID, and BAD levels in tumor tissues were determined by Western blot. Data are presented as mean ± SD, with individual data points overlaid. Statistical comparisons were performed using one-way ANOVA; n = 3 independent experiments. ^**^p<0.01, ^***^p<0.001 *vs*. sh-NC.

Human PBMC and 22RV1 cells were injected into nude mice to construct the 22RV1 xenograft nude mice model with human immune system, followed by the injections of sh-NC or sh-*TBX21*. Levels of cytotoxic proteins, including STAT1, TNF-α, GZMB, and Perforin in tumor tissues were sharply enhanced by sh-*TBX21* injection (**Figure 7A**). Moreover, anti-inflammatory factors, including TGF-β, IL-10, IL-13, and Arg1 in tumor tissues were notably downregulated by sh-*TBX21* injection (**Figure 7B**). Furthermore, levels of apoptosis related proteins in tumor tissues, including Bim, BID, and BAD, were sharply boosted by sh-*TBX21* injection (**Figure 7C**).

## Discussion

In malignant tumors, the function of *TBX21* is complex and variable, and its expression levels and activity may vary depending on the type and stage of cancer. It is shown by some studies that *TBX21* exhibits tumor-suppressing effects in certain types of cancer, slowing down tumor progression by inhibiting cell proliferation and promoting apoptosis. For instance, in colorectal cancer, *TBX21* inhibits the proliferation of tumor cells and promotes their apoptosis by regulating the ARHGAP29/RSK/GSK3β signaling pathway (23). However, in other types of cancer, *TBX21* may promote tumor growth and metastasis. For example, in lung cancer, *TBX21* affects the progression of the tumor and the prognosis of patients by promoting the self-renewal and survival ability of lung cancer stem cells through the activation of the IL-4 signaling pathway (24). Herein, similar to expressions of *TBX21* in cutaneous melanoma reported by Zhang (25), *TBX21* was strikingly upregulated in PCa tissues based on RNA-seq, the upregulation of which was verified in tumor tissues from PCa patients and PCa cell lines. Moreover, by silencing *TBX21*, the *in vitro* and *in vivo* growth of PCa cells were dramatically repressed, along with enhanced apoptosis in tumor cells, implying that *TBX21* functioned as an oncogene in PCa.

Treg and helper T cells 17 (Th17) play complex and critical roles in the tumor immune microenvironment. Inhibitory cytokines such as IL-10 and TGF-β are secreted by Treg cells, which suppress the immune system’s attack on tumors, thereby promoting immune evasion and progression of tumors (10). The activity of other immune cells such as CD8^+^ T cells and NK cells is inhibited by Treg, reducing the immune surveillance of tumor cells (26, 27). Furthermore, Treg cells can also inhibit apoptosis and enhance the survival ability of tumor cells by direct contact with tumor cells (28). Conversely, Th17 cells promote the inflammatory response of tumors by secreting pro-inflammatory cytokines such as IL-17, which may promote tumor growth and metastasis (29). However, the role of Th17 cells in tumor immunity has a double-edged sword effect, and they may activate the immune system to fight tumors. The balance between Treg and Th17 cells in the tumor immune microenvironment has an important impact on the progression of tumors and the effectiveness of treatment (30). Studies have shown that regulating the ratio and function of Treg and Th17 cells may provide new strategies for tumor treatment. For example, reducing the number of Treg cells or inhibiting their function may enhance the immune system’s attack on tumors, and regulating the activity of Th17 cells may inhibit tumor growth (31–33). Herein, in tumor-bearing mice, the proportion of Th17 cells was markedly enriched by silencing *TBX21*, while the proportion of Treg cells was dramatically repressed by silencing *TBX21*, implying that *TBX21* might enhanced the immunosuppressive function in PCa.

In the immune system, cells such as Treg primarily exert immunosuppressive function by targeting CD4^+^ and CD8^+^ T cells (34). CD4^+^ T cells, also known as T helper cells, constituting a specialized category within the lymphocytic population, exert a critical function within the immunological reaction by aiding the activity of other immune cells and orchestrating the body’s defense against pathogens (35). CD8^+^ T cells are key effector cells in the immune system, which directly kill tumor cells by expressing cytotoxic molecules such as Perforin and Granzyme B (GZMB) (36). TNFα is a critical cytokine secreted by CD8^+^ T cells, which not only promote apoptosis of tumor cells but also regulate the tumor microenvironment and enhance the anti-tumor response of other immune cells (37). TNFα promotes the death of tumor cells by inducing programmed necrosis (38). GZMB, as a member of the granzyme family, enters the interior of tumor cells to activate apoptotic pathways, directly leading to the death of tumor cells. These molecules work together to enhance the tumor-killing ability of CD8^+^ T cells (39). Herein, although the proportion of CD4^+^ T and CD8^+^ T cells, as well as PD-1/Tim-3/Lag-3 highly expressed CD4^+^ T and CD8^+^ T cells in tumor tissues was minorly changed by sh-*TBX21*, TNFα/GZMB highly expressed CD8^+^ T cells were markedly enriched by silencing *TBX21*, along with remarkably enhanced STAT1/TNFα/GZMB/Perforin levels and reduced anti-inflammatory cytokine levels in tumor tissues. These data implied that *TBX21* might suppress the anti-tumor property of CD8^+^ T cells, which was not associated with the proliferation of CD8^+^ T cells or the immune checkpoint in CD8^+^ T cells. Moreover, in conformity with Xiong’s report (40), the proportion of Treg cells in activated CD4^+^ T cells was sharply reduced by sh-*TBX21*-transfected PCa cells, indicating that *TBX21* might limit the anti-tumor property of CD8^+^ T cells via enhancing the proportion of Treg cells. In addition, the proportion of Treg cells was changed by sh-*TBX21*-transfected PCa cells, not by directly silencing *TBX21* in CD4^+^ T cells, implying that PCa cells might induce the immunosuppressive microenvironment by *TBX21*, possibly via directly interacted with receptors in CD4^+^ T cells or release vesicles supplemented with *TBX21*.

There are several limitations in the present study. First, human PBMC–reconstituted nude mice were used to mimic the human immune system, which may not fully recapitulate the complexity of native immune interactions. Second, PBMCs were obtained from a single healthy donor to minimize inter-donor variability in immune composition. Although all experiments were independently repeated to ensure reproducibility, future studies involving multiple donors will help to strengthen the generalizability of these findings. Third, the sample size of RNA-seq and xenograft experiments was relatively limited, and further validation in larger and clinically relevant cohorts will be needed. Finally, the precise mechanism by which tumor cell–derived *TBX21* influences T-cell differentiation and function remains to be elucidated and will be explored in subsequent investigations.

Collectively, *TBX21* not only facilitated the growth of PCa cells, but also induced the immunosuppressive microenvironment in PCa by enhancing the proportion of Treg cells, which might be a promising potential target for PCa treatments.

## Data Availability

The raw data supporting the conclusions of this article will be made available by the authors, without undue reservation.

## Supplementary material

Supplementary Figure 1 Gating strategy for identifying CD4^+^ T-cell subsets (CD4^+^Foxp3^+^ Tregs and CD4^+^IL-17A^+^ Th17 cells) by flow cytometry.
