# Supplementary figures and images for: TBX21 correlates with an immunosuppressive tumor microenvironment and Treg/Th17 imbalance in prostate cancer
Source: Front Oncol. 2026 Jan 7;15:1701148. doi: 10.3389/fonc.2025.1701148 (PMC12819308; doi:10.3389/fonc.2025.1701148)

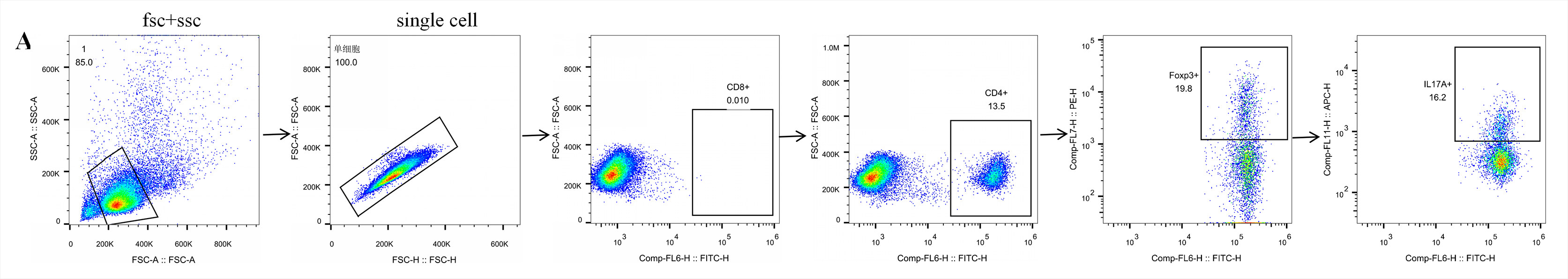

Supplement: Supplementary Figure 1 — Gating strategy for identifying CD4+ T-cell subsets (CD4+Foxp3+ Tregs and CD4+IL-17A+ Th17 cells) by flow cytometry. [file Image1.tif]
